# Ethacrynic acid targets GSTM1 to ameliorate obesity by promoting browning of white adipocytes

**DOI:** 10.1007/s13238-020-00717-7

**Published:** 2020-05-12

**Authors:** Zhaomeng Cui, Yang Liu, Wei Wan, Yuyan Xu, Yehui Hu, Meng Ding, Xin Dou, Ruina Wang, Hailing Li, Yongmei Meng, Wei Li, Wei Jiang, Zengxia Li, Yiming Li, Minjia Tan, Dengke K. Ma, Yu Ding, Jun O. Liu, Cheng Luo, Biao Yu, Qiqun Tang, Yongjun Dang

**Affiliations:** 1 Key Laboratory of Metabolism and Molecular Medicine, The Ministry of Education, Department of Biochemistry and Molecular Biology, Shanghai Medical College, Fudan University, Shanghai, 200030 China; 2 Institute of Stem Cell Research and Regenerative Medicine, Institutes of Biomedical Sciences, Fudan University, Shanghai, 200030 China; 3 State Key Laboratory of Drug Research, Shanghai Institute of Materia Medica, Chinese Academy of Sciences, Shanghai, 201203 China; 4 Department of Physiology and Biophysics, School of Life Sciences, Fudan University, Shanghai, 200438 China; 5 Henan Sesame Research Center, Henan Academy of Agricultural Sciences, Zhengzhou, 450002 China; 6 College of Traditional Mongolian Medicine, Inner Mongolia Medical University, Mongolia, 010110 China; 7 State Key Laboratory of Bio-organic and Natural Products Chemistry, Shanghai Institute of Organic Chemistry, Chinese Academy of Sciences, Shanghai, 200032 China; 8 Department of Endocrinology and Metabolism, Huashan Hospital, Fudan University, Shanghai, 200040 China; 9 Department of Physiology, Cardiovascular Research Institute, University of California San Francisco, San Francisco, USA; 10 Department of Pharmacology and Molecular Sciences, The Johns Hopkins University School of Medicine, Baltimore, MD USA

**Dear Editor,**

Obesity is caused by an imbalance between energy intake and expenditure, and has become a global epidemic with over 650 million adults affected. Adipose tissues in mammals are composed of white adipose tissue (WAT) and classical brown adipose tissue (BAT), and their balance is highly related to the occurrence of obesity. The browning of white adipocytes results in “beige” or “brite” adipocytes, which appear functionally similar to classical brown adipocytes, and can be detected in WAT deposits of animals that have been exposed to cold or other inducers (Fu et al., 2015).

Accumulating evidence has emphasized the role of brown and beige adipocytes in counteracting obesity by dissipating chemical energy as heat (Kajimura et al., 2015). This process is reported to be mediated by uncoupling protein 1 (UCP1), which is abundant within the inner mitochondrial membrane of brown and beige adipocytes (Nowack et al., 2017). UCP1 functions as an uncoupler of oxidative phosphorylation by increasing the permeability of the inner mitochondrial membrane that leads to a dissipation of the proton gradient. Several transcriptional regulators act as powerful activators for recruitment of brown adipocytes in WAT, such as PRDM16, and PGC1α (Ohno et al., 2012; Fu et al., 2015).

Compounds such as celastrol (cela), berberine, and artemisinin have been identified as inducing brown-like adipocytes in WAT (Zhang et al., 2014; Ma et al., 2015; Lu et al., 2016). Despite the beneficial effects in thermogenesis, the direct protein targets and specific molecular mechanisms of these compounds are still illusive. Limited efficacy, diminished specificity, and multiple side effects remain major challenges in clinic uses of these compounds. Identifying novel compounds and clarifying direct specific targets for activating beige adipocytes are of great significance.

In previous work, we reported that artemether can induce browning of adipocytes (Lu et al., 2016). In the same study, we also found that ethacrynic acid (Edecrine, EA) (Fig. 1A) activates the expression of *Ucp1* during adipogenesis of 3T3-L1 and mesenchymal cell line C3H10T1/2 (Fig. S1A and S1B). We found that EA treatment led to the smaller lipid droplets (Fig. 1B), higher mRNA and protein levels of UCP1 (Figs. 1C and S1C). Subsequently, we examined the metabolic profile elicited by EA *in vivo*. Mice maintained on high fat diet (HFD; 60% calories from fat) were treated with EA (5 mg/kg) by intraperitoneal injection for 1 week, and then sacrificed with cold exposure. Surprisingly, mice with EA administration showed significantly reduced body weight (Fig. S1D) and improved cold resistance (Fig. 1D). After cold exposure, iWAT were separated and consumption of oxygen was measured. EA significantly increased the oxygen consumption rate (OCAR) in iWAT (Fig. 1E), implying augmented energy expenditure with EA administration. Meanwhile, iWAT of EA administered mice displayed distinctly smaller lipid droplets (Fig. 1F) and increased UCP1 level (Fig. 1F–H), as well as other browning related genes, including *Pgc1α*, *Prdm16*, *Cd137*, *Tmem26*, and particularly *Tbx1* (Fig. 1G), which is considered as a strong marker distinguishing beige adipocytes from either white adipocytes or brown adipocytes (Roh et al., 2018). In addition, mRNA levels of *Pgc1α* and *Prdm16* were detected to increase in BAT with EA treatment, but the mRNA level of *Ucp1* remained unchanged (Fig. S1E). Taken together, these results strongly indicate a distinct effect of EA on enhancing the browning of iWAT induced by cold exposure.

**Figure 1:**
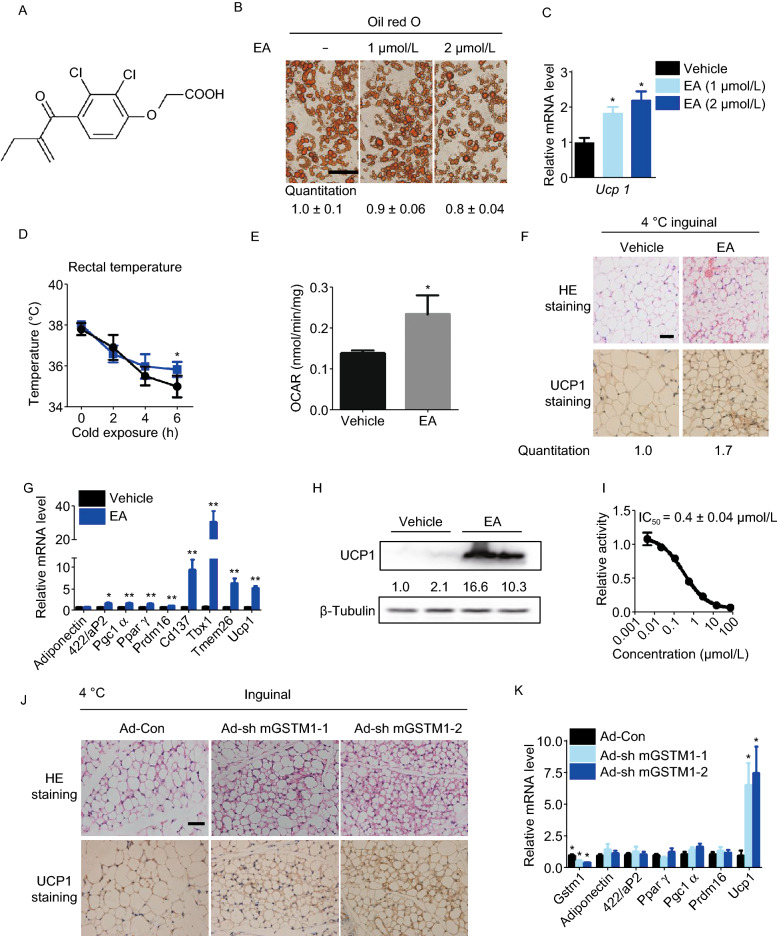

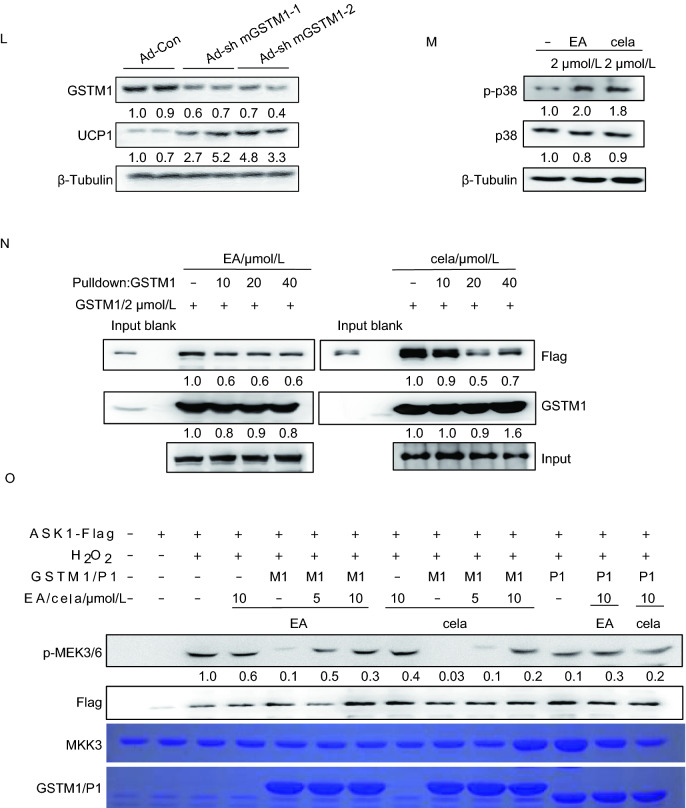
**Ethacrynic acid and celastrol promote browning through GSTM1-ASK1-p38 MAPK pathway**. (A) Chemical structure of ethacrynic acid. (B) Oil red O staining at microscopic levels in C3H10T1/2 cells with or without EA treatment during adipogenesis. Average size of lipid droplets in cells were quantified and showed below the figure. Scale bar 50 μm. (C) Transcriptional levels of Ucp1 in C3H10T1/2 with or without EA treatment during adipogenesis. (D) Rectal body temperature of control and EA-treated mice maintained on HFD during cold exposure (4 °C); *n* = 6 per group. (E) Oxygen consumption rates of inguinal adipose tissues in mice with or without EA treatment for 1 week; *n* = 5 per group. (F) Representative HE staining and IHC staining of UCP1 in the inguinal adipose tissue of HFD-fed mice after treatment with EA for 1 week and then exposed to 4 °C for 8 h. The UCP1 positive area were quantified and showed below. (G) Transcriptional levels of browning-relevant genes in iWAT of HFD-fed mice after EA administration and cold exposure. (H) UCP1 expression level in iWAT of HFD-fed mice after treatment with EA for 1 week exposed to 4 °C for 8 h. (I) Effect of EA on GSTM1 catalytic activity. (J) Representative IHC staining of UCP1 and HE staining with or without GSTM1 knockdown in iWAT of mice with cold exposure at 4 °C for 8 h, *n* = 5. Scale bar, 50 µm. The UCP1 positive area were quantified and showed below. (K) mRNA expression of related genes with or without GSTM1 knockdown in iWAT of mice with cold exposure at 4 °C for 8 h, *n* = 5. (L) Western blot analysis of UCP1 levels with or without GSTM1 knockdown in iWAT of mice with cold exposure at 4 °C for 8 h, *n* = 5. (M) Protein levels of p-p38, and p38 in mature C3H10T1/2 with cela or EA treatment for 3 h. (N) *In vitro* effects of EA/cela on the interaction between GSTM1 and ASK1, performed in duplicate. (O**)** 293T cells were transfected with pcDNA3.1-ASK1-FLAG and treated with H_2_O_2_ (2 mmol/L, 20 min) after 36 h of transfection. The cell lysates were subsequently subjected to immol/Lunoprecipitation with anti-FLAG antibody. The immunopellets were then evaluated for ASK1 activity with immunocomplex kinase assay in the presence of GSTM1 (2 mmol/L) and indicated concentrations of cela and EA. Unless specifically stated, values are presented as mean ± s.e.m from three independent experiments and *P < 0.05, **P < 0.01, ***P < 0.001 compared to control groups, as determined by student’s *t* test

EA is one of the most potent diuretic agents. By binding to the Na^+^-K^+^-2Cl-co-transporter (NKCC2) in the ascending loop of Henle, EA inhibits sodium reabsorption, by a mechanism similar to furosemide (FS) (Milne et al., 2007) Besides, EA is generally known as an inhibitor of glutathione-S-transferases (GSTs) (Mary Schultz et al., 1997). However, the potential role of NKCC2 or GSTs in iWAT browning remains unknown. We began by investigating the function of NKCC2 by treating C3H10T1/2 cells with FS, which is an inhibitor of NKCC2. As shown in Fig. S2A–C, FS did not lead to neither morphological changes of lipid droplets nor increased expression of browning related genes, suggesting the browning effect of EA is most likely not related to NKCC2.

We next asked whether the browning effect of EA is related to GSTs. Cytosolic GST proteins are grouped into of seven classes, including GST alpha (GSTA), mu (GSTM), pi (GSTP), omega (GSTO), kappa (GSTK), zeta (GSTZ) and theta (GSTT). Expression levels of GSTM1 and GSTP1 were much higher than any other classes of GST isoenzymes in iWAT of mice (Fig. S2D). We confirmed that the enzymatic activity of GSTM1 was inhibited by EA *in vitro* (Fig. 1I). The effect of GSTM1 or GSTP1 on browning of adipocytes was investigated with siRNA knockdown, C3H10T1/2 differentiated into brown-like adipocytes typified by small plurilocular lipid droplets and enhanced UCP1 expression upon only disruption of GSTM1 (Fig. S2E–G). When Cl316, 243, a highly selective β3 adrenergic receptor agonist, was used to induce browning-like features during C3H10T1/2 differentiation, the protein level of GSTM1 was slightly reduced while no obvious changes were detected in GSTP1 level (Fig. S2H). Using primary cultured cells from stromal vascular fractions (SVFs) of mice iWAT, we demonstrate that GSTM1 knockdown in SVFs during adipogenesis caused a typical morphology of brown adipocytes and upregulated the brown adipocyte-specific genes like *Ucp1*, *Pgc1α* and *Pparγ* as well (Fig. S2I–K). All these data suggest that inhibition of GSTM1, but not GSTP1, promoted browning of adipocytes. We then found that mRNA levels of GSTM1 were highest in epididymal white adipose tissue (eWAT), followed by iWAT, and lowest in BAT (Fig. S2L and S2N). Although *Gstm2* and *Gstm6* were highly expressed in adipose tissues, knockdown of GSTM2 or GSTM6 during C3H10T1/2 adipogenesis did not enhance *Ucp1* expression which further proved the specific effect of GSTM1 on browning (Fig. S2L and S2M). Subsequently, we decreased iWAT-specific GSTM1 expression in mice using adenoviral delivery. GSTM1 knockdown in iWAT exerted distinct morphological alterations, and the expression levels of UCP1 were upregulated in iWAT with GSTM1 knockdown (Fig. 1J–L). Taken together, these results demonstrate that GSTM1 knockdown during either cell adipogenesis or in iWAT of mice elicits browning. Moreover, we further downregulated GSTM1 by siRNA during C3H10T1/2 adipogenesis, and there was no additive effect of EA on UCP1 activation (Fig. S2O), implying that the effect of EA on browning was mediated by GSTM1.

Celastrol was reported in combating obesity by inducing iWAT browning (Ma et al., 2015), and also can directly disturb the enzymatic activity of GSTs *in vitro* (Zhou et al., 2016). To further confirm that GSTM1 can be a target for induction of browning, we validate the effect of cela on GSTM1 and iWAT browning. As expected, cela treatment induce browning during adipogenesis of C3H10T1/2 cells (Fig. S3A–C). We then confirmed the interaction between cela and GSTM1 *in vitro* through affinity purification assays (Fig. S3D), thermal shift assay (Fig. S3E–G), and enzyme activity assay (Fig. S3H). Cela displayed non-competitive kinetic inhibition of GSTM1 (Fig. S3I). Moreover, we attempted to determine the co-crystal structure of the complex. Although we could not co-crystallize cela with GSTM1, we did successfully obtain a co-crystal structure of cela with mouse GSTP1. GSTP1 functions as a homo-dimeric enzyme, as shown in Fig. S3J, and cela was found to associate with GSH binding pocket of GSTP1 in a dynamic manner (As shown in Fig. S3K, there are two boundary cela binding patterns). Three dimensional structure of mouse GSTM1 was built using SWISS-MODLE with the model PDB 6GSV, and a similar dynamic binding mode of cela with GSTM1 was subsequently built using Maestro (Fig. S3L). The residue M35, M109 formed boundary constrains (Fig. S3L) and the mutation of M35 or M109 by introducing steric hindrance would possibly interfere this dynamic process. The hydrophobic interaction of W8 and V10 with aromatic group of cela played key roles in the binding process. Thus, we obtained GSTM1 mutants W8A, V10S, V10T, M35R and M109R. By conducting microscale thermophoresis (MST) measurements, we found that all mutants showed obvious decreased binding affinity to cela (Fig. S3M). However, the mutants W8A, V10S, and V10T dramatically lost enzymatic activity of GSTM1 (Fig. S3N and S3O). Interestingly, the binding of EA with GSTM1 seemed to be a less dynamic manner, independent of residue M35 or M109 when we aligned the complex of EA with human GSTP1 (PDB 3SGG) and cela with GSTM1 (Fig. S3P). Taken together, these results illustrated that cela directly interacts with GSTM1 and thus exerts its function in induction of iWAT browning (Video S1).

The results above demonstrated that GSTM1 is a potential target for both EA and cela in regulating browning of white adipocytes; however, the downstream effects of GSTM1 inhibition remained unclear. GSTM1 has been shown to inhibit MAP kinase pathway by suppressing the kinase activity of ASK1 (apoptosis signal-regulating kinase 1) in non-stressed cells due to its sequestration via protein-protein interactions (Cho et al., 2001). Given that ASK1-p38 MAPK signaling is critical for activation of beige adipocytes (Dorion et al., 2002), we hypothesized that EA and cela might prevent the interaction between GSTM1 and ASK1, thereby relieving inhibition of ASK1. To test this hypothesis, we first determined the effect of EA and cela on phosphorylation of p38, which is a downstream kinase of ASK1. Both EA and cela could activate p38 MAPK in C3H10T1/2 cells (Fig. 1M). Furthermore, GSTM1 knockdown during adipogenesis of 3T3-L1 led to increased phosphorylation of p38 (Fig. S4A). FLAG-tagged ASK1 was produced in HEK293T cells and incubated with purified GSTM1 in the presence of EA and cela before immunoprecipitation. The result demonstrates that the interaction between GSTM1 and ASK1 is attenuated with either EA or cela treatment (Fig. 1N). Next, we asked whether ASK1 activity was affected by either EA or cela in the presence of GTSM1. *In vitro* kinase assays were carried out and GSTM1, but not GSTP1, dramatically inhibited the phosphorylation of MEK3/6 by ASK1, which was dose-dependently reversed by both EA and cela (Fig. 1O). These data indicated that EA and cela activate ASK1 by disrupting its interaction with GSTM1. Besides, knockdown of ASK1 (Fig. S4B) attenuated the browning effect induced by EA and cela during C3H10T1/2 adipogenesis (Fig. S4C and S4D). Collectively, these data demonstrate that both EA and cela bind to GSTM1 and in turn activate the ASK1 signaling pathway, thereby inducing browning during adipogenesis.

To demonstrate the effect of EA and cela on adiposity and related metabolic disorders *in vivo*, mice were maintained on HFD for 8 weeks, along with intraperitoneal injection of EA or cela. Administration of EA and cela both led to a significant decrease in the body weight of mice (Figs. 2A, 2B and S5A). After dissection, mice treated with EA and cela had less fat, especially in the inguinal and gonadal adipose tissues (Fig. 2C and 2D). Triglycerides and blood glucose were significantly lower in mice treated with EA and cela, while total cholesterol (TC) remained unchanged (Fig. S5B). Besides, mice with EA or cela treatment showed improved glucose tolerance and insulin sensitivity (Fig. 2E and 2F). EA treatment could improve the liver metabolic damage caused by obesogenic stress slightly, cela may cause mild hepatotoxicity (Fig. S5C and S5D). In addition, oxygen consumption (VO_2_) and energy expenditure (EE) was greater in mice treated with cela and EA compared with controls (Fig. 2G and 2H), with CO_2_ production (VCO_2_) not changed (Fig. S5E). The level of physical activity was reduced after EA or cela administration (Fig. S5F). There was also a trend towards increased food intake in EA/cela treated mice, which was statistically insignificant (Fig. S5G). Besides, administration of both of EA and cela led to a stronger adaptation upon cold exposure to maintain body temperature (Fig. 2I). The expression of related genes involved in fat browning were upregulated (Fig. 2J and 2K), and also lipid droplet size was decreased in mice treated with EA and cela respectively (Fig. 2L). We ruled out the effect of EA on obese mice steming from its diuretic effect. When we exposed mice to HFD for 7 weeks, along with intraperitoneal injection of EA, or cela, as well as FS, FS administration did not lead to decrease in the body weight and fat tissues of diet-induced obese mice (Fig. S5H–K). The urine output of FS treated mice was more than that of NC and EA treated groups (Fig. S5L). There was no significant difference in the mean arterial pressure between the treatment groups (Fig. S5M).

**Figure 2:**
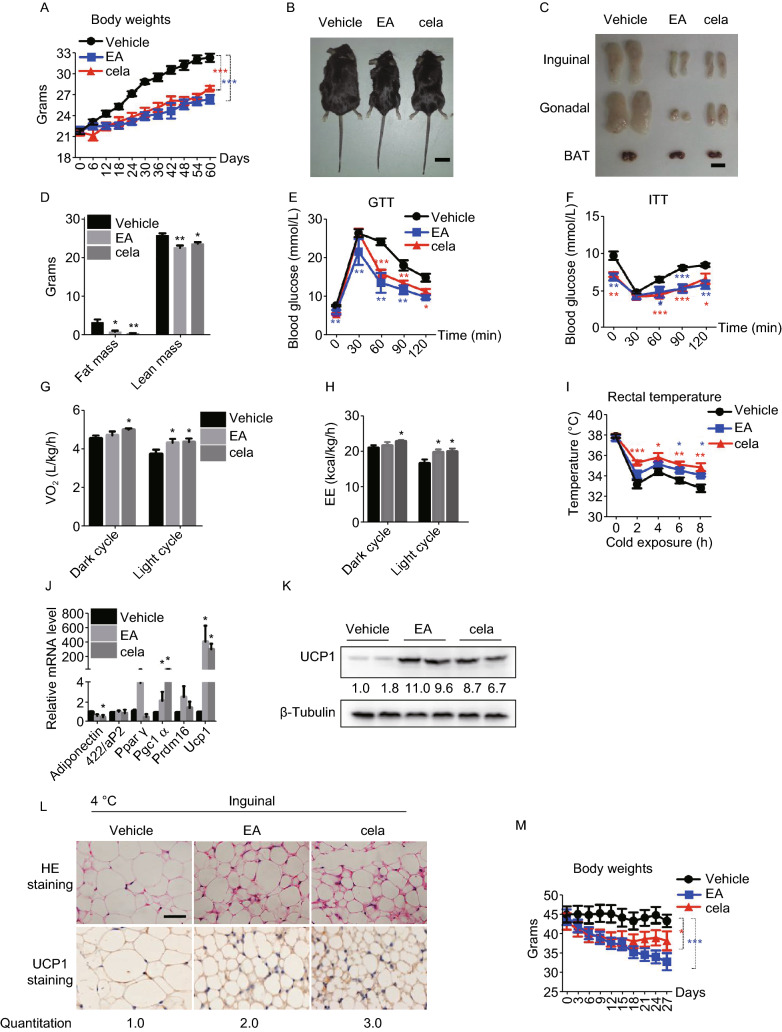

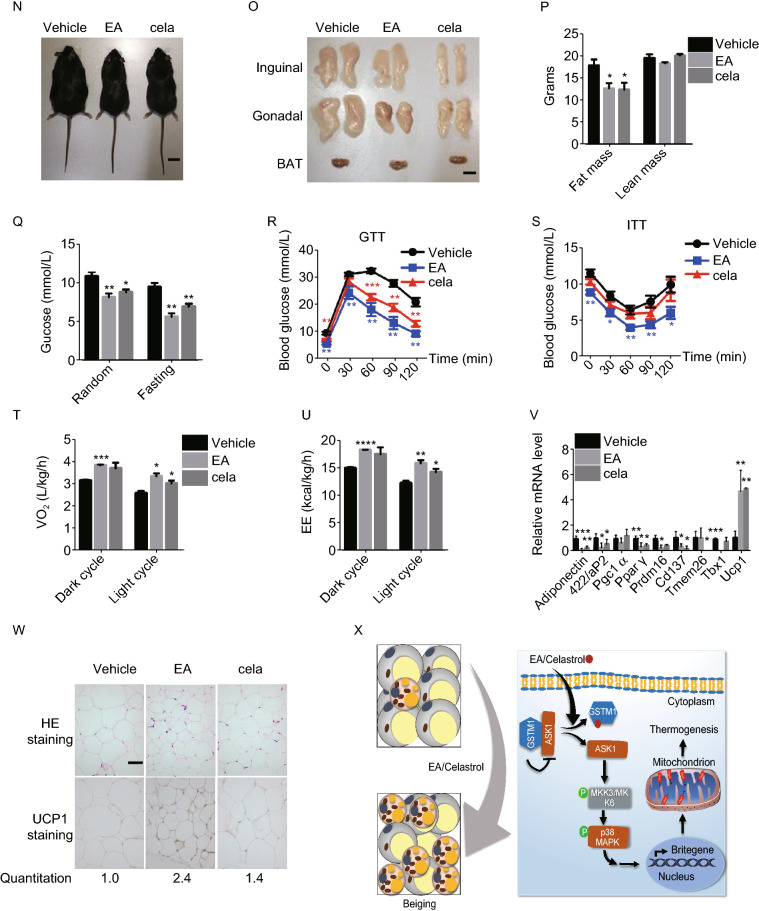
**Ethacrynic acid and celastrol ameliorate adiposity and related metabolic dysfunctions in obese mice**. (A–L) Analysis of mice on high fat diet, either with or without intraperitoneal (i.p.) administration of EA or cela at doses of 5 mg/kg/day and 80 µg/kg/day, respectively, for 8 weeks, including: (A), Body weights; (B and C), Physical builds and the morphology of adipose tissues; Scale bar, 1 cm. (D), Body composition; (E and F), GTT and ITT; (G), Oxygen consumption levels; (H), Energy expenditure; (I), Rectal body temperature measurement during cold exposure at 4 °C for 8 h; (J), qPCR analysis of the expression level of related genes in iWAT of mice with cold exposure at 4 °C for 8 h. (K), UCP1 expression in iWAT of mice with or without EA and cela treatment after cold exposure at 4 °C for 8 h; (L), Representative H&E and UCP1 staining of inguinal WAT with cold exposure after treatment of EA and cela, Scale bar, 50 μm. The UCP1 positive area were quantified and showed below. (M–W) Analysis of mice with DIO, either with or without intraperitoneal (i.p.) administration of EA or cela at doses of 5 mg/kg/day or 80 µg/kg/day, respectively, for 4 weeks, including: (M), Body weights; (N and O), Physical builds and the morphology of adipose tissues; Scale bar, 1 cm. (P), Body composition; (Q), Random blood glucose and fasting blood glucose; (R and S), GTT and ITT; (T), Oxygen consumption levels in 24 h; (U), Energy expenditure in 24 h; (V), qPCR analysis of related genes in iWAT of mice with cold exposure at 4 °C for 8 h; (W), Representative H&E and UCP1 staining in iWAT of mice after cold exposure at 4 °C for 8 h, scale bar, 50 μm. The UCP1 positive area were quantified and showed below. (X), Schematic representation of the molecular mechanisms of EA and cela in enhancing thermogenesis of iWAT of mice. All data are presented as mean ± s.e.m (*n* = 6**–**7) and *P < 0.05, **P < 0.01, ***P < 0.001 compared to control groups, as calculated by Student’s *t* test

To further examine the therapeutic effects of EA and cela on adiposity and related metabolic lesions, mice at 6–8 weeks were maintained on HFD for 8 weeks to construct the model for obesity induced by diet (DIO), which is closest to human obesity. EA and cela were subsequently injected intraperitoneally into DIO mice for 4 weeks. Both EA and cela-administered groups showed reduced body weights and fat tissues (Figs. 2M–P and S5N), improvement in carbohydrate metabolism (Fig. 2Q–S) and energy expenditure (Fig. 2T–U, S5O and S5P). The food intake was not changed in the two groups (Fig. S5Q). Finally, expression of *Ucp1* was upregulated in DIO mice treated with EA and cela (Fig. 2V and 2W), with a corresponding obvious decrease in the size of lipid droplets (Fig. 2W). Furthermore, administration of EA and cela led to a significant decrease in the body weight of ob/ob mice (Fig. S5R and S5S). EA treatment in ob/ob mice showed improved glucose tolerance, lipid metabolism and liver damage (Fig. S5T and S5V). However, neither EA nor cela treatment ameliorated insulin tolerance (Fig. S5U). Overall, these data illustrate that both EA and cela can improve adiposity of mice maintained on HFD and ob/ob genetic defect mice and show potential therapeutic activity in the treatment of adiposity and related metabolic syndrome.

In conclusion, our study shows that EA can promote browning of white adipose tissue and subsequently promote thermogenesis. EA administration in HFD-fed mice exerted positive effects on metabolic dysfunctions including decreased body weight, improved glucose sensitivity, insulin sensitivity, and the ability to preserve body temperature when exposed to cold conditions. Both EA and cela function by targeting GSTM1, which facilitates the increase of ASK1 kinase activity. This is followed by elevated phosphorylation of p38, resulting in brown adipocyte-selective gene expression including Ucp1, which is essential for dissipating energy in brown adipocytes (Fig. 2X).

While our data suggest that browning of white fat tissue induced by GSTM1 knockdown is partly due to the elevated kinase activity of ASK1, we cannot rule out that other regulators may be also partially involved in iWAT browning after GSTM1 knockdown. In addition, our study did not focus on the downstream mechanism by which the GSTM1-ASK1-p38 cascade induces UCP1 expression, and the detailed mechanism remains to be resolved.

GSTM1-mediated regulation of fat tissue adds a new dimension to its known role in metabolism and cellular homeostasis. Effective clinical intervention of obesity based on enhanced thermogenesis is promising yet not realized. We identify GSTM1 as a critical regulator of adipocyte browning and as a target of EA and cela, underscoring therapeutic potential of EA and cela in mitigating obesity and related metabolic imbalance and morbidities in humans.

## Footnotes

We would like to thank Haixin Yuan for providing plasmids mentioned in “Methods”, and we also would like to thank Yun Liu and Bo Wen for kindly sharing their Instruments. We thank Dr. Sarah Head for critical reading and editing of the manuscript.

Y.D. initiates the project, Y.D., Q.T., B.Y., C.L. and J.L designed and supervised the project; Z.C. and Y.L. did experiments and analyzed and prepared data; W.W., Y.H., J.H., and Y.D. contributed to protein structure and computational calculation. Z.C., Y.L., Y.X., M.D., X.D., and Z.L. performed animal experiments. R.W. and W.L. contributed to the chemical synthesis. The manuscript was written by Z.C., Y.L., and Y.D. All authors read and contributed to the final manuscript. This work was supported by the National Key Research and Development Program of China (No. 2018YFC0310900), the National Natural Science Foundation of China grants (31270830, 21572038 and 21877016 to Y.D 81730021 to Q.-Q.T. 81625022, 91853205 to C.L. and K. C. Wong Education Foundation to C.L.), the National Basic Research Program of China (973 Program) (2014CBA02004 to M.T), the Development Fund for Shanghai Talents, Fund of State Key Laboratory of Bioorganic and Natural Products Chemistry, and Fund of State Key Laboratory of Drug Research, Chinese Academy of Science (SIMM1601KF-08).

Zhaomeng Cui, Yang Liu, Wei Wan, Yuyan Xu, Yehui Hu, Meng Ding, Xin Dou, Ruina Wang, Hailing Li, Yongmei Meng, Wei Li, Wei Jiang, Zengxia Li, Minjia Tan, Dengke K. Ma, Yu Ding,Jun O Liu, Cheng Luo, Biao Yu, Qiqun Tang, and Yongjun Dang declare that they have no conflict of interest. All institutional and national guidelines for the care and use of laboratory animals were followed.

The authors declare that all other relevant data supporting the findings of this study is available within the paper and its Supplementary Information files, or from the corresponding authors upon request.

## Electronic supplementary material

Below is the link to the electronic supplementary material.

Supplementary material 1 (PDF 2057 kb)

Supplementary material 2 (MP4 1058 kb)
