# Supplementary material for: Ethacrynic acid targets GSTM1 to ameliorate obesity by promoting browning of white adipocytes
Source: Protein Cell. 2020 May 12;12(6):493–501. doi: 10.1007/s13238-020-00717-7 (PMC8160069; doi:10.1007/s13238-020-00717-7)
Supplement: Supplementary file 1 — Supplementary material 1 (PDF 2057 kb) [file 13238_2020_717_MOESM1_ESM.pdf]

## MATERIALS AND METHODS

Mice

All animal studies were approved by the Institutional Animal Care and Use Committee (IACUC) and were in accordance with NIH guidelines. All mice were provided by the Nanjing Biomedical Research Institute of Nanjing University. Mice were maintained in colony cages at environmental temperature of 22±1°C and humidity of 45±10% using a 12/12 hr light-dark cycle. All mice had free access to water, and food was only withdrawn if required for an experiment.

Adult male C57BL6/J mice at 6-8 weeks of age were divided into 3 groups (n = 6-7mice/group), subjected to 60% HFD (60% energy in kcal from fat, AIN-76A 9G03 Research Diets). For treatments, cela and EA were injected intraperitoneally. To estimate the metabolic parameters of mice with EA and cela treatment, mice were maintained on HFD for 8 weeks, and body weights were recorded every three days throughout the experiment. Body composition was assessed using an NMR analyzer (Bruker). Moreover, energy expenditure, oxygen consumption, food intake and physical activity were measured with CLAMS (Columbus Instruments, Columbus, OH, USA). Parameters such as glucose sensitivity, insulin sensitivity, and cold tolerance were measured at the end of the treatment. At the end of the experiment, tissues of inguinal fat, epididymal fat, brown fat, and liver were collected by dissection, snap-frozen in liquid nitrogen for protein and RNA analysis or fixed in 4% paraformaldehyde for Immunohistochemistry.

RNA extraction and reverse transcription (RT)-qPCR

Total RNA was isolated from tissues and cultured adipocytes using TRIzol lysis reagent (Invitrogen). Complementary DNA was synthesized from 1 μg total RNA using PrimeScript RT Master Mix (TaKaRa) with random primers following the manufacturer's instruction. Real-time quantitative PCR assays were run in duplicate on a LightCycler 480 instrument (Roche) using 2× Syber green PCR Master Mix (Roche Applied Science, Indianapolis, IN). All relative mRNA expression data were calculated by the delta delta Ct method with 18S ribosomal RNA (18s) as the invariant control. Primers used are shown in Supplementary Table 1. Results are presented as means and standard deviations (SD) from three independent experiments.

Western blotting

Proteins were extracted from adipose tissue samples (after flash freezing) or from cultured adipocytes in lysis buffer. In total, 20-30 μg of protein were loaded onto a 10% SDS-PAGE gel after homogenizing in a buffer containing 50 mM Tris-HCl (pH 7.4), 150 mM NaCl, 25 mM β-glycerophosphate, 10% glycerol, 1% Triton X-100, 1 mM DTT, and the protease inhibitor mixture (Leupeptin, Aprotinin, PMSF and Pepstatin A). Proteins were separated by 10% SDS-PAGE and transferred onto nitrocellulose membranes, which were subsequently immunoblotted with specific antibodies. Antibodies used: Rabbit polyclonal anti-UCP1 (Abcam, ab10983); Rabbit monoclonal anti-GSTM1/2/4/5 (Abcam, ab178684); Rabbit polyclonal anti-PRDM16 (Abcam, ab106410); Rabbit polyclonal anti-PGC1α (Abcam, ab54481); Rabbit monoclonal

anti-phospho-p38 MAPK (Thr180/Tyr182) (Cell Signaling Technology, 4511); Rabbit monoclonal anti-p38 MAPK (Cell Signaling Technology, 8690); Rabbit-anti-phospho-MEK-3/6(Ser189/Ser207) (Santa Cruz Biotechnology, sc-8407); Mouse monoclonal anti-Flag (Sigma-Aldrich, F3165); Mouse monoclonal anti-β-Tubulin (Sungene Biotech, KM9003).

C3H10T1/2 differentiation and treatment

Adipogenesis of the immortalized C3H10T1/2 cell line was performed as described(Lu et al., 2016). Two days after reaching confluence (designated day 0), C3H10T1/2 were induced with rosiglitazone and MDI cocktail. EA and cela treatment started at day-2 and continued through the whole process of adipogenesis.

Oil red O staining

0.7 g of oil red O was dissolved in 200 mL of isopropanol and filtered with 0.22μm filters. The solution was diluted with water (3:2). The differentiated adipocytes were washed twice with cold PBS and fixed with 3.7% formalin for 15min, stained with Oil Red O for 4h at room temperature, washed twice with dd$H_2O$ and dried at room temperature (RT), then visualized and photographed.

Histological Analysis and HE staining

Tissues were fixed with 3.7% paraformaldehyde at 4 °C overnight, then embedded in paraffin, sectioned and stained with hematoxylin and eosin (HE) and with UCP1

antibody (ab10983; Abcam) following standard procedures. Immunohistochemistry was done by Shanghai Rui Yu biotechnology Co., Ltd. Histochemical staining and HE staining of different fat deposits were observed with a positive microscope (BX53F, Olympus) using a 40x objective lens.

Isolation and differentiation of SVF cells

Inguinal fat deposits from four-week old male mice are digested with collagenase at 37℃ for 40-45min(Hattori et al., 2016). The cells and medium were aspirated with 1mL pipets every 10 min. The primary cells were filtered through 100μm cell strainer and centrifuged at 700g at room temperature for 5min to collect SVF. The SVF cell pellets were plated and cultured with pre-warmed 20% FBS +DMEM/F12. The following day the SVF cells were washed with pre-warmed DMEM/F12 medium 3 times, and then pre-warmed 20% FBS + DMEM medium was then added to the cells. The cells were then cultured until they were confluent. Two days after reaching confluence, SVF cells were induced to adipocyte with rosiglitazone and MDI cocktail.

Adenoviral injection into Inguinal Fat

Recombinant adenovirus (Ad) for GSTM1 knockdown was generated using the ViraPower Adenoviral Expression System (Invitrogen, Carlsbad, CA), with LacZ recombinant adenovirus as a negative control. The sequences (5' to 3') for shRNAs were shGSTM1-1: CACCGCCAATCTGCCTTACTTGATCGAAATCAAGTAAGGCAGATTGG, and

shGSTM1-2:

CACCGCAGCTCATCATGCTCTGTCGAAACAGAGCATGATGAGCTGC. The recombination and purification of adenovirus were performed as previously described(Liu, 2015). Purified adenovirus was diluted in 50μL saline and was injected bilaterally (s.c.) into the inguinal fat pads of mice (aged 4-6 weeks) for 2 weeks (for a total of 3 times)(Ma et al., 2015). Mice were exposed to cold for 8 h and sacrificed to dissect inguinal white fat. One third of the tissue was fixed with 4% phosphate-buffered polyformaldehyde for histological analysis. The rest of the tissue was subjected to RNA extraction, qPCR analysis, and western blotting.

Glutathione S-transferase Assay and inhibition studies

The activity of GSTM1 was determined in accordance with a previous procedure(Ploemen JHTM, 1996), with 1 mM GSH and 1 mM CDNB as substrates at 25°C in 0.1 M potassium phosphate buffer pH 7.0. A microplate reader (Envision, Perkin Elmer, America) was used to estimate the activity by reading absorbance at 340 nm. For inhibition studies, various concentrations of EA or cela were incubated with GSTM1 for 20 min on ice, then the activity assays were performed.

The kinetic values of GSTM1 for GSH and CDNB were determined by the procedure as follows: enzyme activity was measured at five different concentrations of GSH or CDNB with fixed saturated concentrations of the other substrate. The $K_m$ constant and $V_{max}$ value were calculated from Lineweaver–Burk graphs. Lineweaver–Burk graphs were drawn by using 1/V versus 1/GSH.

Bio-cela Capture Assays

Bio-cela was obtained from amide condensation reaction promoted by HOBt, EDCI, and TEA in anhydrous $CH_2Cl_2$ conditions(Tang et al., 2015). The product was purified by sequential wash, extraction, and column chromatography.

Bio-cela pull-downs were performed as described previously(Low et al., 2005). Purified GSTM1 was preincubated with cela for 40min, followed by incubation with bio-cela for 40min. GSTM1 was captured by streptavidin-coated agarose (Sigma Aldrich) for 1h, followed by 3x 5 min washes with 1 ml of buffer. The agarose was boiled in SDS-PAGE loading buffer for 10min and subjected to 10% SDS-PAGE, followed by Coomassie blue brilliant staining.

Thermal shift assay and isothermal dose-response fingerprint (ITDRF)

Thermal shift assay was performed according to(Ablinger et al., 2013; Jafari et al., 2014). For ITDRF, purified GSTM1 was heated for 3 min at 49.4°C after incubating with different concentrations of cela for 20 min on ice, followed by the transfer of the supernatant into a new Eppendorf tube. The supernatant was boiled for 10min with 1x sample buffer and then subjected to 10% SDS-PAGE and stained with Coomassie blue brilliant.

Crystallization, data collection, and structure determination

Mouse GSTP1 was cloned into the pET series prokaryotic system and expressed by IPTG induction. Recombinant GSTP1 was purified by Glutathione Sepharose-4B,

Mono Q and Superdex 200 Increase columns. Purified protein was concentrated in the following buffer: 50 mM Tris-HCl, pH8.0, 150 mM NaCl, 5mM reduced GSH and 1 mM DTT. Crystals of mouse GSTP1 were generated using the hanging drop method. The purified and concentrated recombinant GSTP1 was mixed with cela in a 1:5 molar ratio and used for crystal screening. Finally, the crystals were grown by mixing 1μL of GSTP1 (10 mg /mL)/cela mixture and 1 μL crystallization solution (26% PEG5000 MME, 100mM MES, pH 6.7 and 200mM ammonium sulfate). The 2μL drop was seeded on a glass slide and inverted over a reservoir containing 0.5 mL of the same crystallization solution and incubated at 20°C. After 5-7 days, crystals grew to a sufficient size and were transferred into a cryogenic buffer (30% PEG5000 MME, 100mM MES, pH6.7, 200mM ammonium sulfate and 20% glycerol) for 15 seconds, and then scooped into a loop and flash frozen in liquid nitrogen. The X-ray diffraction data were collected at 100 K in the beamlines BL17U1 and BL19U1, Shanghai Synchrotron Radiation Facility, Chinese Academy of Sciences.

Diffraction images were indexed and processed by HKL2000 19. The structure of GSTP1 was solved by molecular replacement with the MolRep program from the CCP4 crystallography package using PDB ID code 3O76 as the search model. The model was adjusted by COOT and Phenix. The related figures were drawn by PyMOL.

Fluorescent MST measurement

The purified recombinant proteins (20 μM each) were exchanged into HEPES buffer (20 mM HEPES, pH 7.4, 150 mM NaCl), and then labeled as the protocol of protein labeling kit RED-NHS (Nanotemper, #L001). The MST experiment was performed using Monolith NT.115 instrument (NanoTemper Technologies). We prepared 12 concentrations of dilution series to begin with 2000 μM cela or 5000 μM EA, and preformed 2-fold serial dilutions. Subsequently, labeled GSTM1 and its mutants M35R, M109R, V10T, V10S, and W8A (final concentration between 2.6 and 31 μM) were mixed with various concentrations of candidate compounds in the reaction buffer containing 20 mM HEPES, pH 7.4, 150 mM NaCl, 5%DMSO and 0.1% Surfactant P20. After 5 min incubation at room temperature, the mixtures were aspirated into capillaries for measurement. The MST data were then collected under 20% infrared laser power and 20% light-emitting diode power. The data were analyzed by Nanotemper analysis software (1.5.41) and Thermoporesis + T Jump method was used to determine the $K_D$.

Protein pulldown assay

To explore the interaction between GSTM1 and ASK1, pcDNA3.1-ASK1-Flag was transfected into HEK293T cells. After 36 h, cells were lysed with lysis buffer (20 mM Tris-HCl (pH 7.4), 150 mM NaCl, 0.5% Triton X-100, 10mM NaF, and 0.5% sodium deoxycholate) with protease inhibitors (Leupeptin, Aprotinin, PMSF and Pepstatin A)(Cho, 2001), followed by treatment with indicated concentrations of EA or cela for 1h, and then incubated with with Ni-NTA agrose resin for 1h (GenScript,

L00250-100). The beads were then washed three times with lysis buffer. The immunoprecipitates were separated by 10% SDS-PAGE for Western blotting with rabbit anti-GSTM1 antibody and mouse-anti-Flag antibody.

Kinase activity assay

Assessment of the kinase activity of ASK1 was performed as previously described(Dorion, 2002). pcDNA3.1-ASK1-Flag was transfected into HEK293T cells. After 48hr, cells were stimulated with $H_2O_2$ (2mM) at 37°C for 20min. Cells were extracted in lysis buffer containing 20 mM Tris-HCl, pH 7.4, 150 mM NaCl, 1 mM $Na_3VO_4$, 10 mM NaF, 0.2% NP40, 0.5% deoxycholate and the protease inhibitor mixture (Leupeptin, Aprotinin, PMSF and Pepstatin A). The extracts were centrifuged at 12,000 rpm for 15 min at 4 °C. The clarified supernatants were incubated with indicated concentrations of purified GSTM1 and cela or EA for 1h, followed by incubation with anti-FLAG M2 agarose for 2h with rotating. The kinase activity assay was performed in 30μL of kinase buffer (20 mM Tris-HCl, pH 7.4, 20 mM $MgCl_2$, and 100μM ATP) at 30°C, and was stopped by addition of 1x SDS-PAGE loading buffer. The samples were then subjected to western blotting.

Respiration rate measurement

Mice with or without EA administration were exposed to 4°C for 8 h with free access to water and food. Inguinal adipose tissues were then dissected, snipped and subjected to OxyTrace+ (v1.0.48, Hansatech Instruments) to detect oxygen consumption rates.

GTT and ITT

For the glucose tolerance testing (GTT), mice treated for 8 weeks were placed in a clean cage with water, and injected with D-glucose (2 mg/g body weight, intraperitoneally) following an overnight fasting (16 h). Tail vein blood glucose levels were monitored every 30min post-injection. For insulin tolerance testing (ITT), mice were injected with insulin (Humulin; 1 U kg−1, Sigma-Aldrich, intraperitoneally) after fasting for 4h, and tail vein blood glucose levels were measured every 30min after injection.

Cold exposure and rectal temperature measurements

To test resistance to cold exposure, mice were exposed to 4 °C for 8 h with free access to water and food. Rectal temperature was monitored using a rectal thermometer (PhysiTemp Instruments) every 2h.

Serum Parameters Detection

Blood samples were collected by retrorbital bleeding methods using heparinized capillary tubes. Blood samples were then transferred to Eppendorf tubes and centrifuged within 30 min of collection to separate plasma. Glucose, triglycerides, cholesterol, LDL, HDL, ALT, and AST levels were assayed using the automatic biochemical analyzer (cobas c 311; Roche).

Body Composition Measurements

Live mice were placed into a thin-walled plastic cylinder, and body composition tests were assessed by the NMR analyzer (the minispec Live Mice Analyzer (LF50), Bruker) according to the manufacturer's instructions.

Metabolic Studies(CLAMS)

Two-month-old mice were maintained individually in a metabolism chamber (Comprehensive Lab Animal Monitoring System, CLAMS) with free access to food and water for 72 hours. Mice were housed for 24 hours for adaption. Metabolic parameters including energy expenditure, $O_2$ consumption, $CO_2$ production, respiratory exchange ratio and total locomotor activity was recorded at 10 min intervals in a standard light-dark cycle (light 7:00-19:00 and dark 19:00-7:00) at 25 °C. Respiratory quotient is the ratio of carbon dioxide production to oxygen consumption ($VO_2$). $RER=VCO_2/VO_2$. Energy expenditure was calculated as the product of the calorific value of oxygen ($3.815*VO_2 + 1.232*VCO_2$)(Lee et al., 2016).

Quantification and statistical analysis

All data are expressed as mean values ± SEM. All figures and statistical analyses were generated using GraphPad Prism 6.0. N indicates the number of animals per group or number of independent experiments. The statistical significance of differences between two groups was assessed using two-tailed Student's t tests. The statistical

significance of differences among more than two groups was assessed using one-way ANOVAs with Sidak's multiple comparison tests.; $p < 0.05$ was considered significant. The level of significance was set at *$p < 0.05$; **$p < 0.01$; ***$p < 0.001$.

**REFERENCES**


Ablinger, E., Leitgeb, S., and Zimmer, A. (2013). Differential scanning fluorescence approach using a fluorescent molecular rotor to detect thermostability of proteins in surfactant-containing formulations. International journal of pharmaceutics *441*, 255-260.

Cho, S., Lee, YH., Park, H., Ryoo, K., Kang, K., Park, J., Eom, SJ., Kim, MJ., Chang, TS., Choi, SY., Shim, J. (2001). Glutathione S-Transferase Mu Modulates the Stress-activated Signals by Suppressing Apoptosis Signal-regulating Kinase 1. The Journal of biological chemistry *276*, 12749–12755.

Dorion, S., Lambert, H., Landry, J. (2002). Activation of the p38 signaling pathway by heat shock involves the dissociation of glutathione S-transferase Mu from Ask1. The Journal of biological chemistry *277*, 30792-30797.

Hattori, K., Naguro, I., Okabe, K., Funatsu, T., Furutani, S., Takeda, K., and Ichijo, H. (2016). ASK1 signalling regulates brown and beige adipocyte function. Nature communications *7*, 11158.

Jafari, R., Almqvist, H., Axelsson, H., Ignatushchenko, M., Lundback, T., Nordlund, P., and Martinez Molina, D. (2014). The cellular thermal shift assay for evaluating

drug target interactions in cells. Nat Protoc *9*, 2100-2122.

Lee, J., Liu, J., Feng, X., Salazar Hernandez, M.A., Mucka, P., Ibi, D., Choi, J.W., and Ozcan, U. (2016). Withaferin A is a leptin sensitizer with strong antidiabetic properties in mice. Nat Med *22*, 1023-1032.

Lee, JH., Koo, T.H., Yoon, H., Jung, H.S., Jin, H.Z., Lee, K., Hong, Y.-S., and Lee, J.J. (2006). Inhibition of NF-κB activation through targeting IκB kinase by celastrol, a quinone methide triterpenoid. Biochemical pharmacology *72*, 1311-1321.

Liu, Y., Ge, X., Dou, X., Guo, L., Liu, Y., Zhou, SR., Wei, XB., Qian, SW., Huang, HY., Xu, CJ., Jia, WP., Dang, YJ., Li, X., Tang, QQ. (2015). Protein Inhibitor of Activated STAT 1 (PIAS1) Protects Against Obesity-Induced Insulin Resistance by Inhibiting Inflammation Cascade in Adipose Tissue. Diabetes *64*, 4061-4074.

Low, W.K., Dang, Y., Schneider-Poetsch, T., Shi, Z., Choi, N.S., Merrick, W.C., Romo, D., and Liu, J.O. (2005). Inhibition of eukaryotic translation initiation by the marine natural product pateamine A. Molecular cell *20*, 709-722.

Lu, P., Zhang, F.C., Qian, S.W., Li, X., Cui, Z.M., Dang, Y.J., and Tang, Q.Q. (2016). Artemisinin derivatives prevent obesity by inducing browning of WAT and enhancing BAT function. Cell Research *26*, 1169-1172.

Ma, X.R., Xu, L.Y., Alberobello, A.T., Gavrilova, O., Bagattin, A., Skarulis, M., Liu, J., Finkel, T., and Mueller, E. (2015). Celastrol Protects against Obesity and Metabolic Dysfunction through Activation of a HSF1-PGC1α Transcriptional Axis. Cell metabolism *22*, 695-708.

Ploemen JHTM, M.L., Wormhoudt LWCJNM. (1996). In vitro inhibition of rat and human glutathione S-transferase isoenzymes by disulfiram and diethyldithiocarbamate. Biochemical pharmacology *52*, 197-204.

Tang, W.J., Wang, J., Tong, X., Shi, J.B., Liu, X.H., and Li, J. (2015). Design and synthesis of celastrol derivatives as anticancer agents. European journal of medicinal chemistry *95*, 166-173.

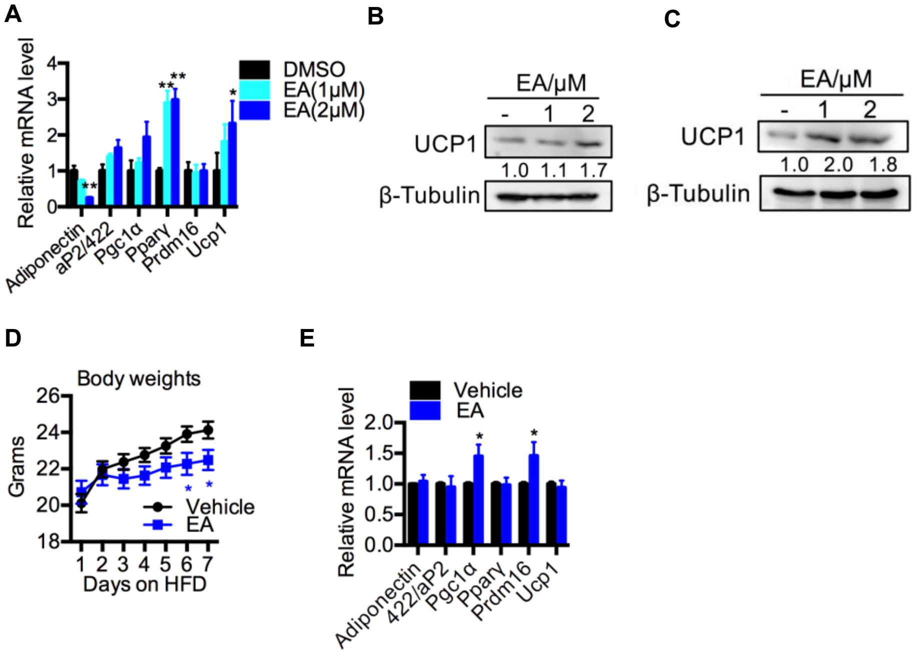


**Supplemental Figure 1 EA exerts browning effects in iWAT of mice. A** Transcriptional level of Ucp1 and related genes during adipogenesis of 3T3-L1. **B** UCP1 expression level of control and EA treated 3T3-L1 cell line. **C** UCP1 expression levels in C3H10T1/2 cells with or without EA treatment during adipogenesis. **D** Body weights of control and EA-treated mice maintained on HFD for 1 week；n=6 per group. **E** Transcriptional levels of browning-relevant genes in BAT of HFD-fed mice after treatment with EA for 1 week. Unless specifically stated, values are presented as mean ± s.e.m from three independent experiments and *P < 0.05, **P < 0.01, *** P<0.001 compared to control groups, as determined by student’s t test.

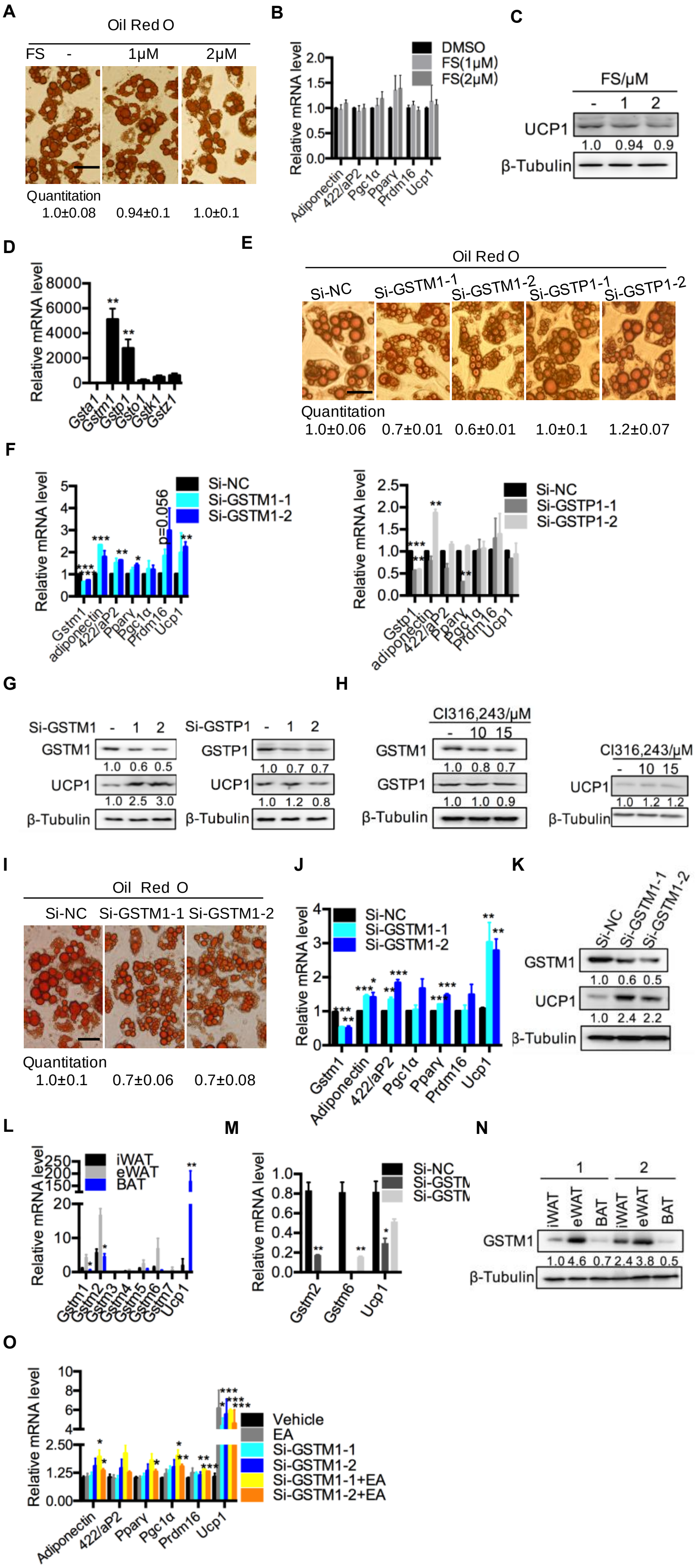
A
Oil Red O
FS - 1μM 2μM
Quantitation
1.0±0.08 0.94±0.1 1.0±0.1
B
DMSO
FS(1μM)
FS(2μM)
Relative mRNA level
Adiponectin 422/aP2 Pgc1α Pparγ Prdm16 Ucp1
C
FS/μM
- 1 2
UCP1
1.0 0.94 0.9
β-Tubulin
D
Relative mRNA level
Gsta1 Gstm1 Gstp1 Gsto1 Gstk1 Gstz1
E
Oil Red O
Si-NC Si-GSTM1-1 Si-GSTM1-2 Si-GSTP1-1 Si-GSTP1-2
Quantitation
1.0±0.06 0.7±0.01 0.6±0.01 1.0±0.1 1.2±0.07
F
Si-NC
Si-GSTM1-1
Si-GSTM1-2
p=0.056
Relative mRNA level
Gstm1 adiponectin 422/aP2 Pparγ Pgc1α Prdm16 Ucp1
Si-NC
Si-GSTP1-1
Si-GSTP1-2
Gstp1 adiponectin 422/aP2 Pparγ Pgc1α Prdm16 Ucp1
G
Si-GSTM1 - 1 2
GSTM1
1.0 0.6 0.5
UCP1
1.0 2.5 3.0
β-Tubulin
Si-GSTP1 - 1 2
GSTP1
1.0 0.7 0.7
UCP1
1.0 1.2 0.8
β-Tubulin
H
CI316,243/μM
- 10 15
GSTM1
1.0 0.8 0.7
GSTP1
1.0 1.0 0.9
β-Tubulin
CI316,243/μM
- 10 15
UCP1
1.0 1.2 1.2
β-Tubulin
I
Oil Red O
Si-NC Si-GSTM1-1 Si-GSTM1-2
Quantitation
1.0±0.1 0.7±0.06 0.7±0.08
J
Si-NC
Si-GSTM1-1
Si-GSTM1-2
Relative mRNA level
Gstm1 Adiponectin 422/aP2 Pgc1α Pparγ Prdm16 Ucp1
K
Si-NC Si-GSTM1-1 Si-GSTM1-2
GSTM1
1.0 0.6 0.5
UCP1
1.0 2.4 2.2
β-Tubulin
L
iWAT
eWAT
BAT
Relative mRNA level
Gstm1 Gstm2 Gstm3 Gstm4 Gstm5 Gstm6 Gstm7 Ucp1
M
Si-NC
Relative mRNA level
Gstm2 Gstm6 Ucp1
N
1 2
iWAT eWAT BAT iWAT eWAT BAT
GSTM1
1.0 4.6 0.7 2.4 3.8 0.5
β-Tubulin
O
Relative mRNA level
Vehicle
EA
Si-GSTM1-1
Si-GSTM1-2
Si-GSTM1-1+EA
Si-GSTM1-2+EA
Adiponectin 422/aP2 Pparγ Pgc1α Prdm16 Ucp1

**Supplemental Figure 2 GSTM1 modulates the development of brown-like characteristics during C3H10T1/2 and SVF adipogenesis. A** The morphological change in C3H10T1/2 with FS treatment during adipogenesis. Average size of lipid droplets in cells were quantified and showed below the figure. Scale bar, 50μm. **B** mRNA expression of related genes in C3H10T1/2 with FS treatment during adipogenesis. **C** Protein levels of UCP1 in C3H10T1/2 with FS treatment during adipogenesis. **D** mRNA expression analysis of Gst supergene family in iWAT. **E** Oil red O staining at microscopic levels in C3H10T1/2 cell line with GSTM1 or GSTP1 knockdown. Average size of lipid droplets in cells were quantified and showed below the figure. Scale bar 50 μm. **F** Transcriptional levels of browning relevant genes during adipogenesis of C3H10T1/2 with GSTM1 or GSTP1 knockdown. **G** UCP1 expression level during adipogenesis of C3H10T1/2 with GSTM1 or GSTP1 knockdown. **H** GSTM1 and GSTP1 protein levels in mature C3H10T1/2 stimulated with Cl316,243. **I** Morphological change in SVF with GSTM1 knockdown during adipogenesis. Average size of lipid droplets in cells was quantified and showed below the figure. Scale bar, 50μm. **J** mRNA expression of related genes in SVF with or without GSTM1 knockdown during adipogenesis. **K** Protein levels of UCP1 in SVF with or without GSTM1 knockdown during adipogenesis. **L** Comparison of the expression levels of GSTM subtypes between iWAT, eWAT, and BAT. **M** Transcriptional levels of Ucp1 during adipogenesis of C3H10T1/2 with GSTM2 or GSTM6 knockdown. **N** GSTM1 expression between iWAT, eWAT, and BAT. **O** mRNA expression levels of related genes in C3H10T1/2 with GSTM1 knockdown or

EA treatment during adipogenesis. Unless specifically stated, values are presented as mean ± s.e.m from three independent experiments and *P < 0.05, **P < 0.01, *** P<0.001 compared to control groups, as determined by student's t test.

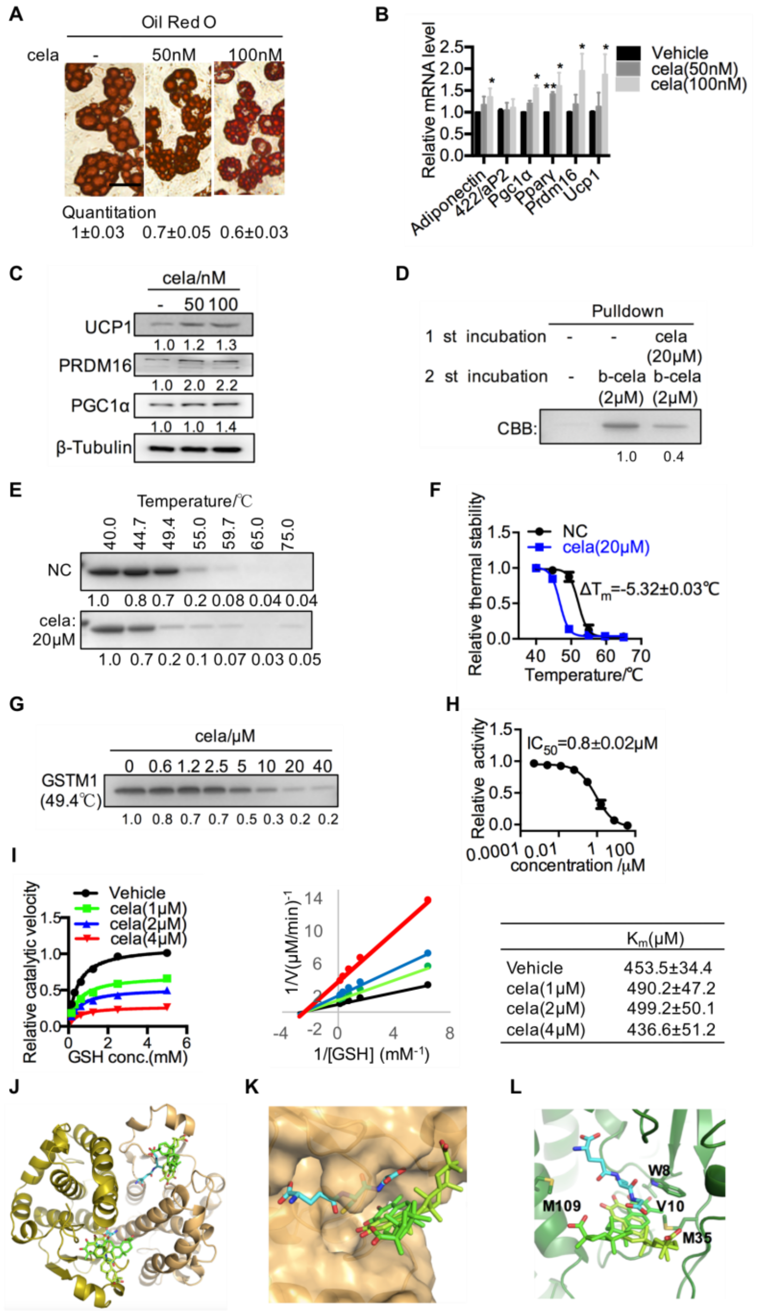


M

| Proteins | $K_d$(μM) |
|---|---|
| GSTM1 | 6.73 |
| GSTM1-W8A | >100 |
| GSTM1-V10S | >100 |
| GSTM1-V10T | >100 |
| GSTM1-M35R | >100 |
| GSTM1-M109R | >100 |

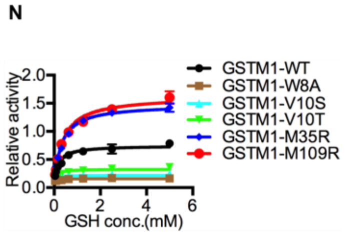


O

| GSTM1 | $K_m$(μM) | $V_m$(μM/min) |
|---|---|---|
| WT | 157.8±23.0 | 746.3±26.1 |
| W8A | 19.5±9.5 | 158.7±4.1 |
| V10S | 31.9±7.5 | 219.6±7.6 |
| V10T | 59.9±11.6 | 328.8±11.8 |
| M35R | 295.8±29.5 | 1479±32.9 |
| M109R | 365.5±37.8 | 1625±47.3 |

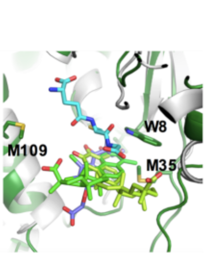

**Supplemental Figure 3 Identification of the interaction between cela and GSTM1 *in vitro*.** **A** Oil red O staining in control and cela treated C3H10T1/2 cell line during adipogenesis. Average size of lipid droplets in cells were quantified and showed below the figure. Scale bar 50 μm. **B** Transcriptional levels of browning relevant genes during adipogenesis of C3H10T1/2 with cela treatment. **C** UCP1 expression level of control and cela treated C3H10T1/2 cell line during adipogenesis. **D** SDS-PAGE analysis of Bio-cela pulldown from recombinant GSTM1 after Coomassie brilliant blue staining. **E-F** Thermal denaturation profiles of recombinant GSTM1 in the presence or absence of cela in potassium phosphate buffer (PH7.0). **G** Isothermal dose-response curve of purified GSTM1 with cela treatment at 49.4℃. **H** Dose-response curve of GSTM1 activity with cela treatment using GSH and CDNB as dual substrates **I** Catalytic activity of GSTM1 with or without cela treatment using GSH and CDNB as dual substrates. **J** Top view of the interaction between GSTP1 and cela. (Bright orange, GSTP1; olive, GSTP1; cyans, GSH; green, cela; limon, cela.) **K** Ribbon diagram of cela bound to GSTP1. (Bright orange, GSTP1; cyans; green, cela; limon, cela.) **L** Surface representation of GSTM1 bound by cela. (Forest, GSTM1; cyans; green, cela; limon, cela.) **M** KD between cela and different point mutations of GSTM1 measured by MST. **N-O** Kinetics of substrate saturation of different point mutations of GSTM1. **P** Different binding pattern of EA and cela with GSTM1, forest, GSTM1; gray, GSTP1 (PDB: 3GSS); cyan, GSH; slate, EA; green and limon, cela.

Unless specifically stated, values are presented as mean ± s.e.m from three independent experiments and $^{*}P < 0.05$, $^{**}P < 0.01$, $^{***}P<0.001$ compared to

control groups, as determined by student's t test.

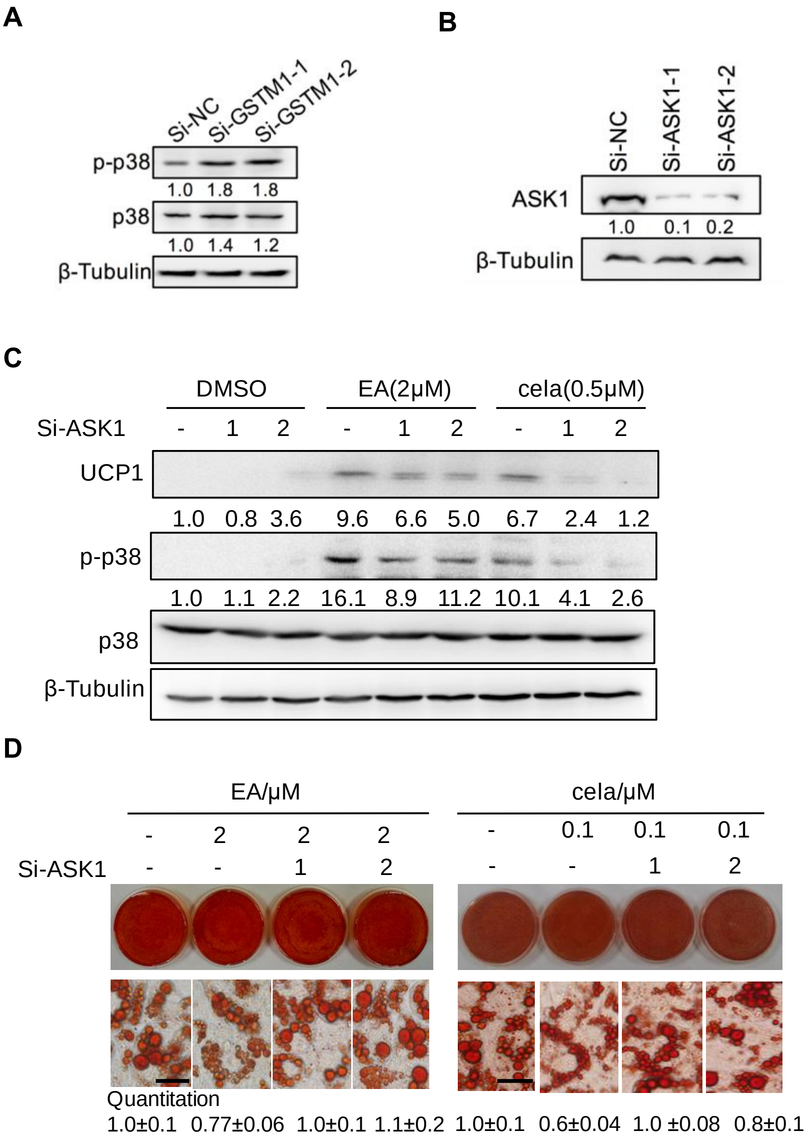


**Supplemental Figure 4 ASK1 debilitated the function of EA and cela in inducing morphology changes and phosphorylation of p38 during differentiation. A** Immunoblotting analysis of phosphorylated-p38 and total p38 with GSTM1 knockdown during C3H10T1/2 adipogenesis. **B** ASK1 knockdown by siASK1. **C** Western blot analysis of p-p38, and total p38 during C3H10T1/2 adipogenesis treated with cela or EA with ASK1 knockdown. **D** Morphology changes induced by EA or cela in C3H10T1/2 with ASK1 knockdown during adipogenesis. Average size of lipid droplets in cells were quantified and showed below the figure. Scale bar, 50μm.

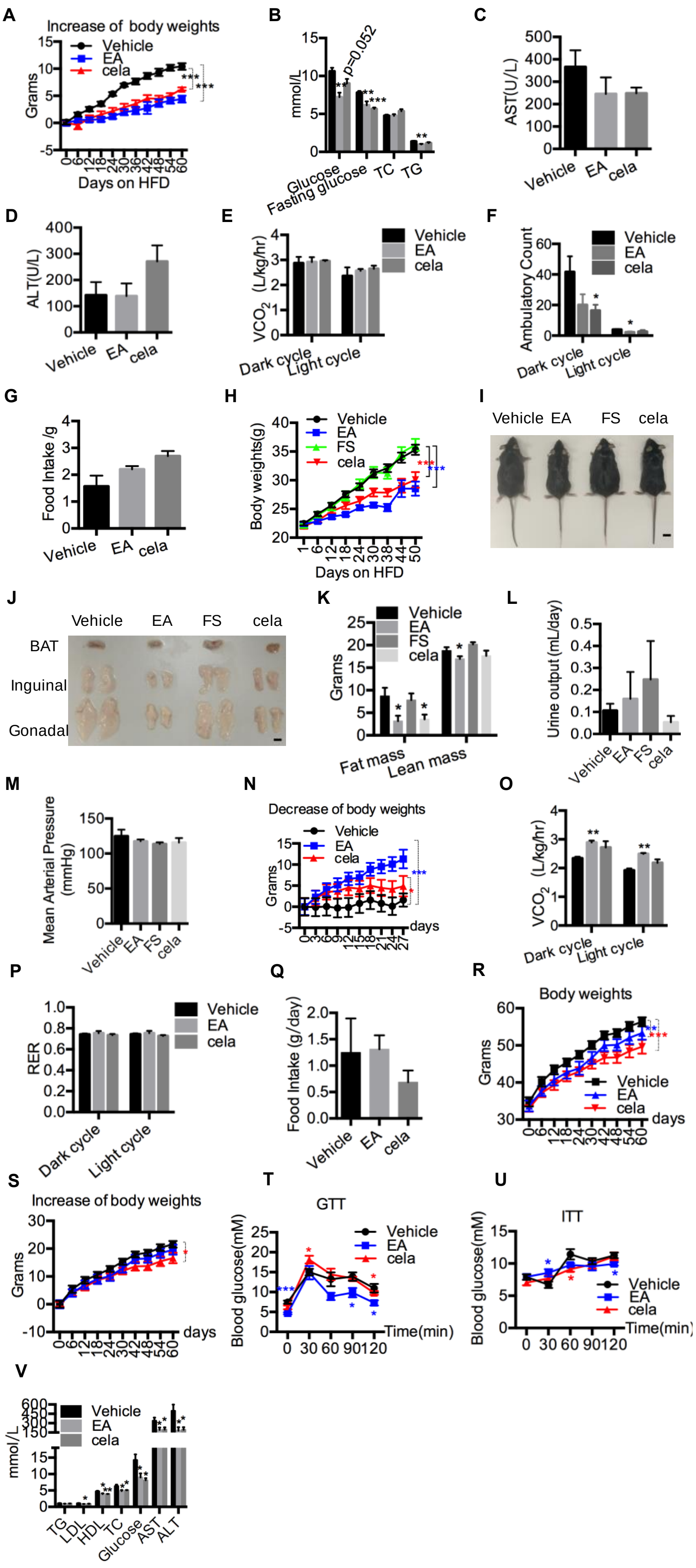
A
Increase of body weights
Vehicle
EA
cela
Grams
Days on HFD
B
mmol/L
p=0.052
Glucose
Fasting glucose
TC
TG
C
AST(U/L)
Vehicle
EA
cela
D
ALT(U/L)
E
VCO2 (L/kg/hr)
Dark cycle
Light cycle
F
Ambulatory Count
G
Food Intake /g
H
Body weights(g)
FS
I
J
BAT
Inguinal
Gonadal
K
Fat mass
Lean mass
L
Urine output (mL/day)
M
Mean Arterial Pressure (mmHg)
N
Decrease of body weights
days
O
P
RER
Q
Food Intake (g/day)
R
Body weights
S
T
GTT
Blood glucose(mM)
Time(min)
U
ITT
V
LDL
HDL
AST
ALT

**Supplemental Figure 5 EA and cela improve obesity and related metabolic disorders in obese mice. A-G** Analysis of mice on high fat diet, either with or without intraperitoneal (i.p.) administration of EA or cela at doses of 5 mg/kg/day and 80μg/kg/day, respectively, for 8 weeks, including: (**A**), Increase of body weights; (**B**), Serum parameters; (**C**), AST level; (**D**), ALT level; (**E**), $VCO_2$; (**F**) Physical activity; (**G**) Food intake. **H-M** Analysis of mice maintained on HFD, either without or administered intraperitoneally(i.p.) with EA or FS or cela at doses of 5 mg/kg/day, 5 mg/kg/day or 80μg/kg/day, respectively for 7 weeks, including: (**H**), body weights; **(I and J**), Physical builds and the morphology of adipose tissues; Scale bar, 1cm; (**K**), Body composition; (**L**), urine output; (**M**), mean arterial pressure. **N-Q** Analysis of mice with DIO, either with or without intraperitoneal (i.p.) administration of EA or cela at doses of 5 mg/kg/day or 80μg/kg/day, respectively, for 4 weeks, including: (**N**), decrease of body weights; **(O),** $CO_2$ production in 24h; **(P),** Respiratory exchange rate, RER; (**Q**), Food intake. **R-V** Analysis of DIO mice, either without or administered intraperitoneally (i.p.) with EA or cela at doses of 5 mg/kg/day or 80μg/kg/day, respectively for 4 weeks, including: **(R),** Body weights; **(S),** Increase of body weights; (**T**), GTT; (**U**), ITT; (**V**), Serum parameters. Unless specifically stated, values are presented as mean ± s.e.m from three independent experiments and *P < 0.05, **P < 0.01, *** P<0.001 compared to control groups, as determined by student's t test.

**Table1． Primers used in RT-PCR assays**

| Primers | Forward sequence | Reverse sequence |
|---|---|---|
| *Gstm1* | GTTCTTGAAGACCATCCCTG AG | GGGCTCAAACATACGGTACTG |
| *Gsta1* | GGGTGGAGTTTGAAGAGAA GT | TGGCGATGTAGTTGAGAATGG |
| *Gsto1* | CCAGATGACCCGTACAAGA AG | GTTCGGAGAGTCTTCCTTTCT C |
| *Gstk1* | CGCATCCTGGAACTCTTCTA C | CCGCAACTGCAGCTTTATATT C |
| *Gstp1* | TGCCATCTTGAGACACCTTG | GTTGGTGTAGATGAGGGTGAC |
| *Gstz1* | GATGAGGTATCCATGGCTGA TG | GTTGATGTGACTGATGGTAGG G |
| *Ucp1* | GGCCCTTGTAAACAACAAAA TAC | GGCAACAAGAGCTGACAGTA AAT |
| *PGC1α* | ACCATGACTACTGTCAGTCA CTC | GTCACAGGAGGCATCTTTGAA G |
| *Adiponectin* | TGTTCCTCTTAATCCTGCCCA | CCAACCT GCACAAGTTCCCTT |
| *422/aP2* | CCTTTGTGGGAACCTGGAA | CTGTCGTCTGCGG TGATT |
| *Pparγ* | ATACATAAAGTCCTTCCCGC TG | GTGATTTGTCCGTTGTCTTTCC |

| | | |
|---|---|---|
| *Prdm16* | CCACCAGCGAGGACTTCAC | GGAGGACTCTCGTAGCTCGAA |
| *18s* | CGGCTACCACATCCAAGGAA | GCTGGAATTACCGCGGCT |
| *Cd137* | ATCTTCAGAGCAGTTCAAGG G | GCTTTGTCCCTTTCCTAAACG |
| *Tbx1* | TGTGGGACGAGTTCAATCAG | TGTCATCTACGGGCACAAAG |
| *Tmem26* | CTCTGGTAGCGGACATTACA C | GAGCAAGGAATAGGGAAGGG |
